# Improving Management of Paediatric Buckle Fracture in Orthopaedic Outpatients: A Completed Audit Loop

**DOI:** 10.7759/cureus.1829

**Published:** 2017-11-08

**Authors:** MN Baig, C Egan

**Affiliations:** 1 Trauma & Orthopaedics, Galway University Hospital

**Keywords:** buckle fracture, nice

## Abstract

Introduction

Paediatric patient bone fractures are the source of a large number of orthopaedic outpatient visits, especially for fracture clinics. The National Institute for Health and Care Excellence (NICE) guideline NG38 provides guidance on assessing and managing non-complex fractures, such as buckle (i.e., torus) fractures in paediatric patients.

Objective

We retrospectively audited outpatient records of children younger than 12 years presenting with distal radius buckle fractures for May and June 2017. We compared our practice against the NICE guideline standards. We made certain changes in our practice and then repeated the exercise prospectively for two months from July 15 to September 15, 2017.

Material and Methods

We identified 31 patients who fit our inclusion criteria. After instituting changes based on the NICE guidelines, the number of children included in the prospective data collection was 33 patients.

Results

For the 31 children treated according to our older protocol, we had 59 outpatient visits, with an average of 1.90 visits for every child. After the NICE-driven changes were made to our management, 33 patients were treated in 39 visits with an average of 1.2 visits per child.

Conclusion

Introducing NICE guidelines allowed for considerable improvement in the management and treatment of paediatric patient bone fractures. It is important to fully implement the NICE guidelines not only in fracture clinics but also in other departments, such as accident and emergency departments.

## Introduction

Treating paediatric patient bone fractures makes up a sizeable portion of orthopaedic fracture clinic activities. Paediatric patient fractures make up approximately 25% of the paediatric injuries we treat [1]. Paediatric fractures are referred to the fracture clinics from accident and emergency departments (A&ED) for an expert opinion regarding further management. In many instances, the follow-up orthopaedic fracture clinic visits are not required. We audited distal radius buckle fracture presentations and follow-up visits and then assessed our management against the National Institute for Health and Care Excellence (NICE) guidelines. We adapted our program and then performed a second audit.

The 2016 NICE NG38 guidelines cover non-complex fracture management [2]. These guidelines advocate that if the child has sustained a simple buckle fracture proximal to the distal radius growth plate, the fracture should be treated with a non-rigid cast or a splint [2].

## Materials and methods

We conducted our first audit in April and May 2017. We retrospectively collected the data from 31 children under age 12 who presented to our fracture clinic after referral from A&EDs with a buckle fracture. We also noted subsequent visits to the fracture clinic for treatment of the same condition.

**Figure 1:**
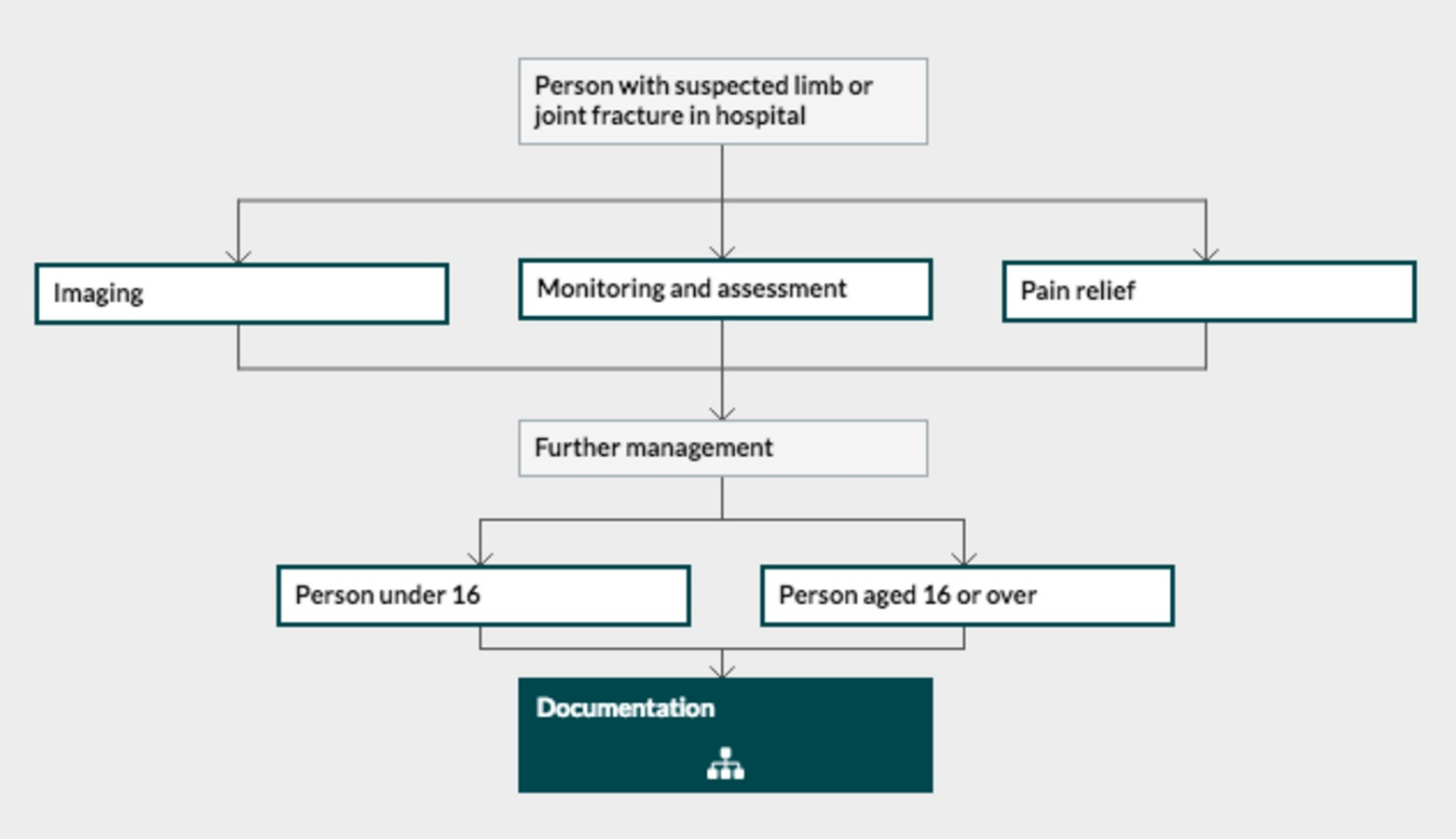
Algorithm from National Institute for Health and Care Excellence (NICE) guideline NG38 NICE fracture management guideline with main steps.

**Figure 2:**
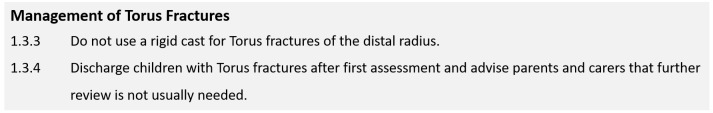
National Institute for Health and Care Excellence (NICE) NG38 management guideline

In June, we adjusted our practice to align with the NICE guidelines by teaching parents about the fracture type, informing them how to remove the cast, and offering them no further visits. Relevant portions of the NICE guidelines that influenced the changes are shown in Figures 1-2.

We then collected data for July and August 2017. There were 33 children under age 12 who presented with buckle fractures to our fracture clinics. We also tracked subsequent visits for the same condition for at least six weeks after the initial visit.

**Figure 3:**
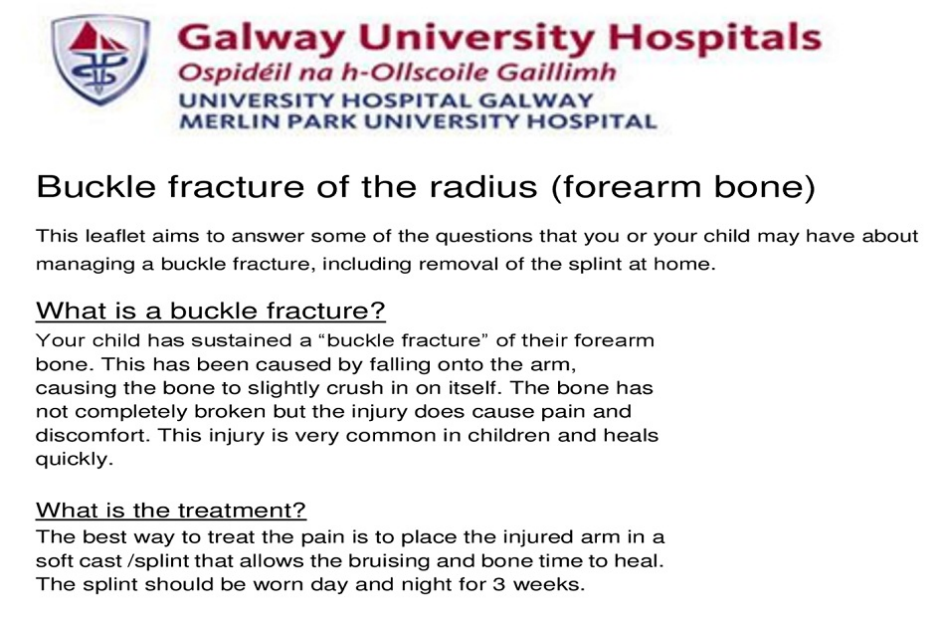
Information leaflet - page 1

**Figure 4:**
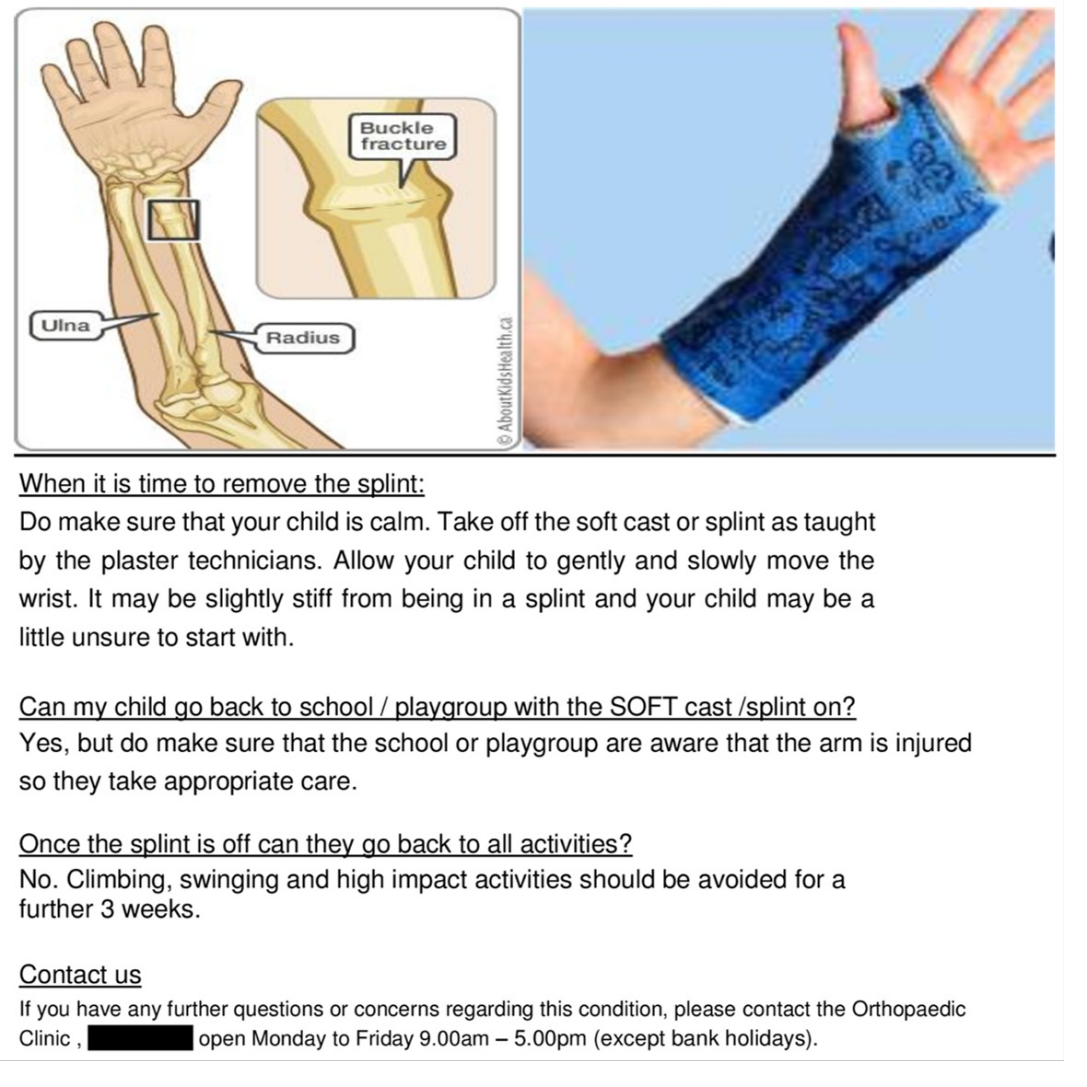
Information leaflet - page 2

To inform parents about the type of fracture and help explain why further visits are unnecessary, we worked with our clinic plaster technicians and nurses and devised a new information leaflet. The plaster technicians calmly instructed the parents on removing the soft cast at home without special equipment or expertise. The information leaflet we designed is shown in Figures 3-4.

## Results

We had no certain protocol for the consistent treatment of buckle fractures for the 31 paediatric patients who presented in April and May. They had 59 total visits over the next few weeks, including their initial presentations. Some of the children came on three subsequent visits, but the average was one additional visit after the initial visit (1.9 visits) (Table 1).

**Table 1:** Patient Visits Table showing two sets of results. First set shows visits in three months from April to May. Second set showing from July to August. A considerable reduction in follow-up visits is visible. OPD: Outpatient department

Months	Number of Patients	Fracture OPD Visits	Average Visits
April to May	31	59	1.9
July to August	33	39	1.2

In the months tracked after implementing the NICE guidelines, we had 33 children presenting in July and August 2017 with 39 total visits, including their initial presentation. The average number of visits had decreased to 1.2 (Table 1).

## Discussion

Implementing the NICE guidelines significantly decreased the number of visits and reduced the workload on fracture clinics which are often overbooked; the doctors are tired from the work and patients are tired due to long wait times [3-4]. While the reduction in visits is relatively small, this demonstrated that smarter and more practical approaches could help control and manage clinical numbers.

This reduction in necessary visits also relieves patients and parents who must make time for the extra visits, which can be hard for children enrolled in school. The most important aspect of reducing visits was educating the parents about the fracture type and how to safely remove the soft cast at home [5].

During our study, we also reviewed the practices of some very good paediatric hospitals with paediatric orthopaedic departments, and they, too, were implementing this practice successfully.

For future studies, we would like to compare clinical outcomes from using a soft cast versus a wrist immobilization splint.

We are the first unit in Ireland to complete a program audit loop for the treatment of paediatric patient buckle fractures, and the results are satisfactory. 

## Conclusions

The adage, “If you always do what you always did, you always get what you always got” applies to medical practices and efforts to improve. Improved practices are not possible without positive intervention. The complete audit loop showed that we improved our practice by following the NICE guidelines. Other institutions can apply this approach to measure the impact of changes in patient management. In our case, we saved time and resources for doctors, clinic staff, and perhaps more importantly, patients.
